# Combination of white matter hyperintensities and Aβ burden is related to cognitive composites domain scores in subjective cognitive decline: the FACEHBI cohort

**DOI:** 10.1186/s13195-021-00877-6

**Published:** 2021-08-17

**Authors:** G. Ortega, A. Espinosa, M. Alegret, GC. Monté-Rubio, O. Sotolongo-Grau, A. Sanabria, JP. Tartari, O. Rodríguez-Gómez, M. Marquié, A. Vivas, M. Gómez-Chiari, E. Alarcón-Martín, A. Pérez-Cordón, N. Roberto, I. Hernández, M. Rosende-Roca, L. Vargas, A. Mauleón, C. Abdelnour, E. Esteban De Antonio, R. López-Cuevas, S. Alonso-Lana, S. Moreno-Grau, I. de Rojas, A. Orellana, L. Montrreal, L. Tárraga, A. Ruiz, M. Boada, S. Valero, FACEHBI group, N. Aguilera, S. Alonso-Lana, M. Berthier, M. Buendia, S. Bullich, F. Campos, P. Cañabate, L. Cañada, C. Cuevas, S. Diego, A. Gailhajenet, P. García, J. Giménez, R. Gismondi, M. Guitart, M. Ibarria, A. Lafuente, F. Lomeña, E. Masip, E. Martín, J. Martínez, M. Moreno, A. Niñerola, A. B. Nogales, L. Núñez, A. Páez, A. Pancho, E. Pelejà, V. Pérez-Grijalba, A. Perissinotti, P. Pesini, S. Preckler, N. Roé-Vellvé, J. Romero, M. I. Ramis, M. Sarasa, M. A. Tejero, M. Torres

**Affiliations:** 1 Fundació ACE, Institut Català de Neurociències Aplicades, Research Center and Memory Clinic, Universitat Internacional de Catalunya, C/ Gran Via de Carles III, 85 bis- 08028 Barcelona, Spain; 2 Networking Research Center On Neurodegenerative Diseases (CIBERNED), Instituto de Salud Carlos III, Madrid, Spain; 3 Departament de Diagnòstic Per La Imatge, Clínica Corachan, Barcelona, Spain

**Keywords:** Amyloid-beta (Aß), Apolipoprotein E, Cognitive composites domain scores, FBB-PET, Magnetic resonance imaging, Subjective cognitive decline, White matter hyperintensities

## Abstract

**Background:**

To explore whether the combination of white matter hyperintensities (WMHs) and amyloid-beta (Aβ) deposition is associated with worse cognitive performance on cognitive composites (CCs) domain scores in individuals with subjective cognitive decline (SCD).

**Methods:**

Two hundred participants from the FACEHBI cohort underwent structural magnetic resonance imaging (MRI), ^18^F-florbetaben positron emission tomography (FBB-PET), and neuropsychological assessment. WMHs were addressed through the Fazekas scale, the Age-Related White Matter Changes (ARWMC) scale, and the FreeSurfer pipeline. Eight CCs domain scores were created using the principal component analysis (PCA). Age, sex, education, and apolipoprotein E (*APOE*) were used as adjusting variables.

**Results:**

Adjusted multiple linear regression models showed that FreeSurfer (*B* − .245; 95% CI − .1.676, − .393, *p* = .016) and β burden (SUVR) (*B* − .180; 95% CI − 2.140, − .292; *p* = .070) were associated with face–name associative memory CCs domain score, although the latest one was not statistically significant after correction for multiple testing (*p* = .070). There was non-significant interaction of these two factors on this same CCs domain score (*p* = .54). However, its cumulative effects on face–name associative performance indicated that those individuals with either higher WMH load or higher Aβ burden showed the worst performance on the face–name associative memory CCs domain score.

**Conclusions:**

Our results suggest that increased WMH load and increased Aβ are independently associated with poorer episodic memory performance in SCD individuals, indicating a cumulative effect of the combination of these two pathological conditions in promoting lower cognitive performance, an aspect that could help in terms of treatment and prevention.

## Background

Scientific evidence and neuropathological studies suggest that cerebrovascular diseases (CVDs) frequently co-occur with Alzheimer’s disease (AD) pathology [1]. Asymptomatic CVDs such as white matter hyperintensities (WMHs) are common in the brains of healthy aging adults and are important characteristics that together with cardio-metabolic risk factors such as hypertension (HTA), diabetes mellitus (DM), insulin resistance, obesity/overweight (OB), and hyperlipidemia (DLP) are associated with cognitive decline [2], increased risk of mild cognitive impairment (MCI) [1–4], and dementia [5, 6] including vascular cognitive impairment (VCI) and AD [7, 8].

Greater amyloid-beta (Aβ) burden has widely been reported as a potential key factor related to deleterious effects on cognition, suggesting that subtle cognitive changes accrue as amyloid amounts increase [3, 4]. Positron emission tomography (PET) has facilitated the early detection of subjects with Aß burden and consequently with a high risk of AD in later life [5, 6]. Additionally, the relationship between magnetic resonance imaging (MRI) measurements of vascular brain injury, such as WMHs, and Aβ burden and cognition has been investigated [7–9], finding that vascular brain injury had a greater influence across all measured cognitive domains and was not related to Aβ burden.

There is increasing evidence that the subjective experience of cognitive decline may reflect an early stage of actual cognitive decline [10, 11]. The term subjective cognitive decline (SCD) has been introduced to refer to these kinds of symptoms [12]. Those self-reported complaints, identified with SCD diagnosis, may present with markers for small vessel disease (SVD), such as WMHs. However, to date, there is little data about WMHs in preclinical AD and their relation to AD biomarkers such as Aβ. Most patients with AD are diagnosed once cognitive impairment is already established, but it is well known that identifying the disease in early stages brings great benefits not only in terms of treatment but also in terms of cost savings and prevention [13]. Thus, there is a growing need for accurate identification of asymptomatic WMH individuals with underlying AD pathology to improve the diagnosis of prodromal and presymptomatic AD. With this study, we are the first to investigate this aspect in combination with Aβ burden in subjects presenting with SCD in a clinical setting.

Hence, with a cross-sectional analysis of 200 participants with SCD from the Fundació ACE Brain Health Initiative (FACEHBI) [14] cohort, we aimed to explore the relationship of WMHs and Aβ deposition with performance on cognitive composites (CCs) domain scores in individuals with SCD, providing answers to the following hypotheses: (1) WMHs and Aβ deposition are associated with poor cognitive performance on specific CCs domain scores and (2) the combined impact of WMH load and Aβ burden is associated with a worse cognitive performance.

## Material and methods

### Subjects

The study was embedded in the FACEHBI project [14] and used a convenience sample of 200 individuals diagnosed with SCD at Fundació ACE (Barcelona, Spain) [15] recruited from the Open House initiative (70%) and the Diagnostic Unit (30%) [16]. SCD was defined as the coexistence of cognitive complaints and a score of ≥ 8 on the Spanish Modified Questionnaire of Memory Failures Every day (MFE-30) [17]. Inclusion criteria were (a) subjects older than 49 years, (b) Mini Mental State Exam (MMSE) ≥ 27 [18, 19], (c) Clinical Dementia Rating (CDR) = 0 [20], (d) performance in the Fundació ACE Neuropsychological Battery (NBACE) [21] within the normal range for age and education, and (e) literate. A further description of the exclusion criteria is provided in Rodriguez-Gomez et al. [14]. All participants underwent neurological and cognitive examinations, including the NBACE and additional neuropsychological tests, a set of self-administered questionnaires, and a battery of multimodal biomarkers that included FBB-PET, brain MRI, apolipoprotein E (*APOE*) genotyping, and an optical coherence tomography (OCT) scan of the retina. All participants gave written informed consent, and the FACEHBI protocol was approved by the ethics committee of the Hospital Clinic i Provincial (Barcelona, Spain) (EudraCT: 2014–000,798–38).

### Cognitive composites construction

All subjects underwent a neuropsychological battery with tests in ten cognitive domains: (1) executive function fluency (Semantic Category Fluency [21], Phonetic (letter) Fluency [21], and Action (verb) Fluency) [22]; (2) executive function processing speed (Trail Making Test part A and part B [23], Automatic Inhibition subtest of the Syndrome Kurtz Test (SKT)) [21]; (3) executive function attention (Digit spans forwards and backwards [21]), Rule Shift Card subtest of the Behavioral Assessment of the Dysexecutive Syndrome (BADS) [23], Similarities (abbreviated to the first 10 items) subtest of Wechsler Adult Intelligence Scale-Third Edition (WAIS-III) [21]; (4) verbal memory (the Word List Learning test from the WMS-III: learning and recall, and recognition) [21]; (5) visual memory (RBANS Figure subtest recall an recognition task) [24]; (6) face–name associative memory (FNAME initial learning (ILN) and cued recall for names (CRN30) [25]); (7) face–occupation associative memory (FNAME initial learning (ILO) and cued recall for occupations (CRO30) [25]); (8) language (Boston Naming Test (BNT) [21], Action Naming Test, Kissing and dancing and Pyramids and palm trees [23, 26], verbal comprehension (Commands item from the ADAS-Cog) [27], Repetition (2 words and 2 sentences) [21]); (9) visuoperception (the Poppelreuter test [21], Luria’s Clock Test [21], 15-Objects Test [21]); and (10) praxis (Block Design (abbreviated, 1 point, without a time bonus) subtest of the WAIS-III [21], RBANS Figure copy) [23].

In order to create the CCs, factorial structure as an aspect of construct validity was first analyzed through the use of a principal component analysis (PCA). The purpose was to determine if there was a single component underlying the neuropsychological items’ variation as the neuropsychological function was intended to be unidimensional. Every PCA was forced to produce a unidimensional factorial solution according to the expected unidimensional neuropsychological function assessed. This yielded eight CCs domain scores as follows: (1) executive function fluency, (2) executive function processing speed, (3) executive function attention, (4) verbal memory, (5) visual memory, (6) face–name associative memory, (7) face–occupation associative memory, and (8) language.

The original variables contributed to the final score in a weighted way, based on the magnitude of the inter-correlations among the variables in the same CCs domain score. The following tests were finally part of each CCs domain score: (1) executive function fluency CCs domain score (Semantic Verbal Fluency [21], Letter Fluency [21], Verb Fluency) [22]; (2) executive function processing speed CCs domain score (Trail Making Test part A and part B [23], Automatic Inhibition subtest of the Syndrome Kurtz Test (SKT) [21]); (3) executive function attention CCs domain score (Digit span forwards and backwards) [21]; (4) verbal memory CCs domain score (The Word List Learning test from the WMS-III: learning and recall, and recognition) [21]; (5) visual memory CCs domain score (RBANS Figure subtest recall and recognition task) [24]; (6) face–name associative memory CCs domain score (FNAME initial learning (ILN) and cued recall for names (CRN30)) [25]; (7) face–occupation associative memory CCs domain score (FNAME initial learning (ILO) and cued recall for occupations (CRO30)) [25]; and (8) language CCs domain score (BNT [21], Action Naming Test [23]).

The stability of the PCA was evaluated by means of Hotelling’s *T* ^2^ test. When a neuropsychological variable obtained a factorial loading < 0.3 in the one-dimension solution, the variable was excluded from the analysis assuming that this variable had a poor empirical contribution to the corresponding inferred cognitive function. The linear function of the original variables from the factorial solution was used as a final standardized domain score for each subject that was identified as a composite in this study. Each cognitive domain corresponded to a CCs domain score that could be later analyzed using standard procedures.

According to this criterion, the following cognitive tests were excluded from the corresponding PCA analyses: BADS [23], Similarities (abbreviated to the first 10 items) subtest of WAIS-III [21], Recognition memory from the WMS-III [21], Kissing and dancing [28], Pyramids and palm trees [26], Verbal comprehension (Commands item from the ADAS-Cog) [21], Repetition (2 words and 2 sentences) [21], and the Poppelreuter-type overlap figures [21], Luria’s Clock Test [21], 15-Objects Test [21], Block Design (abbreviated, 1 point, without a time bonus) subtest of the WAIS-III [21], and RBANS Figure copy [23].

### Neuroimaging acquisition and analysis

All individuals from the FACEHBI study [14] underwent a structural MRI within a 90-day window after the baseline visit. The imaging data were analyzed using the Fundació ACE Pipeline for Neuroimaging Analysis and are available upon request from the corresponding author.

### WMHs and MRI analysis

MRI was performed on a 1.5-T Siemens Magneton Aera (Erlangen, Germany) using a 32-channel head coil. T1-weighted images were acquired using a rapid acquisition gradient-echo 3D MPRAGE sequence with the following parameters: TR 2.200 ms, TE 2.66 ms, TI 900 ms, slip angle 8°, FOV 250 mm, slice thickness 1 mm, and isotropic voxel size 1 × 1 × 1 mm. In addition, all the participants underwent axial T2-weighted, 3D isotropic FLAIR, and axial T2*-weighted sequences. Segmentation of the T1 MRI images was carried out using the FreeSurfer 6.0 recon-all procedure. The FreeSurfer image analysis suite is documented and freely available for download online (http://surfer.nmr.mgh.harvard.edu/). The volume of white matter hypointensities was extracted from the segmentation results. Two visual assessment scales were used to determine WMHs: the Fazekas scale [29] and the Age-Related White Matter Changes (ARWMC) by Wahlund et al. [30] which was constructed for both MRI and CT images. The Fazekas scale divides the white matter into periventricular and deep white matter, and each region is given a grade depending on the size and confluence of the lesions: (a) periventricular white matter (PVWM) (0 = *absent*, 1 = *caps or pencil-thin lining*, 2 = *smooth halo*, 3 = *irregular periventricular signal extending into the deep white matter*) and (b) deep white matter (DWM) (0 = *absent*, 1 = *punctate foci*, 2 = *beginning confluence*, 3 = *large confluent areas*). The ARWMC scale distinguishes five brain regions of the right and left hemispheres, which are scored separately: (1) the frontal area, including the frontal lobe anterior to the central sulcus; (2) the parieto-occipital area, comprising the parietal and occipital lobes; (3) the temporal area, including the temporal lobe (the border between the parieto-occipital and temporal lobes was estimated by a line drawn from the posterior portion of the Sylvian fissure to the trigone areas of the lateral ventricles); (4) the infratentorial area, encompassing the brainstem and cerebellum; and (5) the basal ganglia, including the striatum, globus pallidus, thalamus, internal and external capsules, and insula. The highest score is 30 points.

Image quantification by Fazekas scale and ARWMC scale was performed by two expert radiologists with final consensus, blind to age, sex, race, educational achievement, and diagnostic status, and FreeSurfer segmentation was achieved by an expert physicist.

### FBB-PET and standardized uptake value ratio (SUVR) acquisition

FBB-PET images were acquired in a 90-day window after the baseline visit in a Siemens© Biograph molecular-CT machine. Four FBB-PET scans of 5 min were acquired 90 min after injection of 300 Mbq of florbetaben (18F) radiotracer (NeuraCeq©), administered as a single slow intravenous bolus (6 s/mL) in a total volume of up to 10 mL. The radiotracer was kindly provided by Piramal (currently Life Molecular Imaging; https://life-mi.com/). FBB-PET images were processed with FSL 5.0 suite and co-registered onto structural images. The standardized uptake value ratio (SUVR) was determined as the mean value of the cortical regions segmented on MRI and normalized by the cerebellum gray matter uptake. A cutoff of SUVR = 1.45 was selected as the Aβ positivity criterion—that is, to classify subjects in FBB-PET-positive and FBB-PET-negative groups [31].

### Apolipoprotein E (APOE) genotyping

Genomic DNA was extracted from peripheral blood using the commercially available kit Chemagic system (Perkin Elmer). The *APOE* genotypes were determined with the use of fluorogenic allele-specific oligonucleotide probes (TaqMan assay; Life Technologies, Spain) for rs7412 (Test ID: C____904973_10) and rs429358 (Test ID: C___3084793_20). For the TaqMan assays, PCR and real-time fluorescence measurements were carried out in QuantStudio3 real-time PCR system (Thermo Fisher Scientific, Spain) using the TaqMan Universal Master Mix (ref: 4,364,341, Life Technologies, Spain) methodology according to the manufacturer’s instructions. Polymerase chain reaction was performed as follows: first, a pre-read step for 30 s at 60 °C, a denaturation for 10 min at 95 °C, followed by 40 cycles at 95 °C for 15 s and 60 °C for 1 min, and a post-read stage for 30 s at 60 °C. The genotype was determined using the Genotyping App for Thermo Fisher Cloud by clustering analysis. The laboratory technicians were blinded to other study variables.

### Statistical analyses

In order to see if there was a significant correlation among the three neuroimaging approaches measuring the presence of WMHs, partial correlations with Spearman correlation coefficient were carried out among Fazekas scale, ARWMC scale, and FreeSurfer pipeline. According to neuroimaging approaches, the Fazekas scale, with a high positive asymmetric distribution, was dichotomized into two categories: 0 = *absence* (Fazekas 0–1) and 1 = *presence* (Fazekas 2–3). The FreeSurfer pipeline was subjected to a logarithmic transformation to decrease the range of the variable and log units were used in all analyses because of the non-normal distribution.

To identify possible associations with Aβ burden (SUVR), a Student’s *t* test was used in the comparison with Fazekas condition (*absence/ presence*), and Pearson’s correlation coefficient to explore correlations with ARWMC and FreeSurfer.

Multiple linear regression analysis, including age, sex, educational level, and *APOE* (at least one ε4 allele vs. no ε4 allele) as co-variates, was carried out among every CC domain score and the three neuroimaging scales (Fazekas, ARWMC, and FreeSurfer) and the Aβ burden (SUVR) to analyze the impact of WMHs and SUVR on each CCs domain score. All analyses were corrected for multiple comparisons using Hommel’s method.

Based on the results obtained, we chose those CCs domain scores related to both WMHs and Aβ burden to analyze the second hypothesis.

Finally, multiple regression analyses were performed to assess the combined effect of WHMs (FreeSurfer) and Aβ deposition (SUVR) combination on CCs domain scores adjusting for age, sex, education, and *APOE*. A figure was also done to observe graphically the CCs domain score performance distribution according to WMHs (FreeSurfer) and Aβ deposition (SUVR). High-performance CCs domain scores are with lighter color intensity dots and those with medium performances are represented with medium color intensity dots, while those with low performances are shown with darker color intensity dots.

## Results

### Demographic characteristics of the cohort

From the FACEHBI cohort of 200 individuals with SCD, one individual was excluded due to the acquisition or movement artifacts in the MRI or PET data; therefore, a sample of 199 participants was analyzed. The mean age of the participants was 65.8 ± 7.2 years (range 51–86). 63.5% of the participants were women, and the participants had an average of 14.8 ± 4.6 years (range 3–28) of education. Fifty-two (26%) individuals had at least one *APOE* -ε4 or ε3/ε4 allele. According to the vascular profile, the sample had a median Fazekas score of 1 (range 0–3), a median ARWMC of 1 (range 0–17), and a median WMH volume quantified by FreeSurfer of 1675.25 (range 504.9–12,240.5). The dichotomized Fazekas scale had 70 *absence* subjects and 126 *presence* subjects.

### Associations between WMH load, Aβ burden, and CCs domain scores

First, partial correlation among the three neuroimaging approaches measuring the presence of WMHs showed a significant association (*p* ≤ 0.001) between them.

In the comparison between Fazekas and Aβ burden (SUVR), there was no statistical significant association (*t* =  − 0.60; *p* value = 0.54), neither between ARWMC scale, FreeSurfer pipeline, and Aβ burden (SUVR) (*r* = 0.02; *p* value = 0.70; *r* = 0.09; *p* value = 0.17, respectively).

Multiple linear regression analysis showed a non-significant association between any CCs domain score, Fazekas condition (*presence* and *absence*), and ARWMC, but did show a significant relationship between FreeSurfer and the face–name associative memory CCs domain score (*B* =  − 1.034; 95% CI =  − 1.676, − 0.393; *p* = 0.002). Hence, those subjects with higher WMHs measured by FreeSurfer showed poorer performance in this CCs domain score. Also, the same statistical analysis showed a tendency between Aβ burden (SUVR) and the face–name associative memory CCs domain score (*B* =  − 1.216; 95% CI =  − 2.140, − 0.292; *p* = 0.010) (Table 1).

**Table 1:** Association between CCs domain scores, WMHs, and SUVR

CCs (dependent variable)	Fazekas			ARWMC			FreeSurfer			SUVR		
	B	95% CI	p	B	95% CI	p	B	95% CI	p	B	95% CI	p
**EF-FL**	.030	(− .251, .312)	.832	− .065	(− .450, .320)	.739	− .092	(− .723, .539)	.774	.438	(− .458, 1.334)	.337
**EF-PS**	− .007	(− .266, .252)	.956	.043	(− .313, .399)	.812	.204	(− .375, .783)	.489	.615	(− .208, 1.438)	.142
**EF-AT**	.250	(− .027, .528)	.077	.464	(.080, .848)	.018	.142	(− .49, − .777)	.659	− .549	(− 1.450, .351)	.230
**FN-AM**	− .173	(− .465, .119)	.244	− .164	(− .564, .235)	.418	− 1.034	(− .1.676, − .393)	.002*	− 1.216	(− 2.140, − .292)	.010
**FO-AM**	− .364	(− .651, − .077)	.013	− .392	(− .789, .004)	.056	− .862	(− 1.503, .222)	.009	− .250	(− 1.176, .677)	.596
**VR-M**	− .097	(− .395, .200)	.519	.088	(− .317, .494)	.668	− .070	(− .737, .598)	.837	− .415	(− 1.367, .536)	.390
**VI-M**	.346	(− .159, .452)	.346	.035	(− .389, .459)	.872	− .098	(− .787 − .592)	.837	− 1.313	(− 2.272, − .355)	.008
**Language**	.035	(− .252, .322)	.809	− .004	(− .398, .390)	.984	− .413	(− 1.051, .226)	.204	− .467	(− 1.380, .446)	.314

*CCs* Cognitive Composites domain scores, *EF-FL* Executive Function-Fluencies, *EF-PS* Executive Function Processing Speed, *EF-AT* Executive Function Attention, *FN-AM* Face–Name Associative Memory, *FO-AM* Face–Occupations Associative Memory, *VR-M* Verbal Memory, *VI-M* Visual Memory, *SUVR* Standardized uptake value ratio, *CI* Confidence Interval

Age, years of education, sex, and *APOE* e4 (none vs. one or more alleles e4) were used as adjusting variables. Multiple linear regression analysis between each CCs domain score, Fazekas, ARWMC, FreeSurfer, and SUVR was used; ^*^Adjusted *p* value < .05 after correction for multiple tests (Hommel’s method)

### Combined impact of WMH load and Aβ burden on CCs domain scores

As reported previously, the CCs domain of face–name associative memory appeared as the only domain that was significantly associated with WMH load measured by the FreeSurfer pipeline and with Aβ deposition. The main consideration was to determine whether the combined effect of both variables is associated with an increased effect on the CCs domain score of face–name associative memory.

Multiple regression analyses showed a non-significant interaction effect between these two factors on the face–name associative memory CCs domain score (*p* = 0.54). Hence, no modulation effect of these two pathological conditions can be assumed when explaining face–name associative memory CCs domain score.

**Fig. 1:**
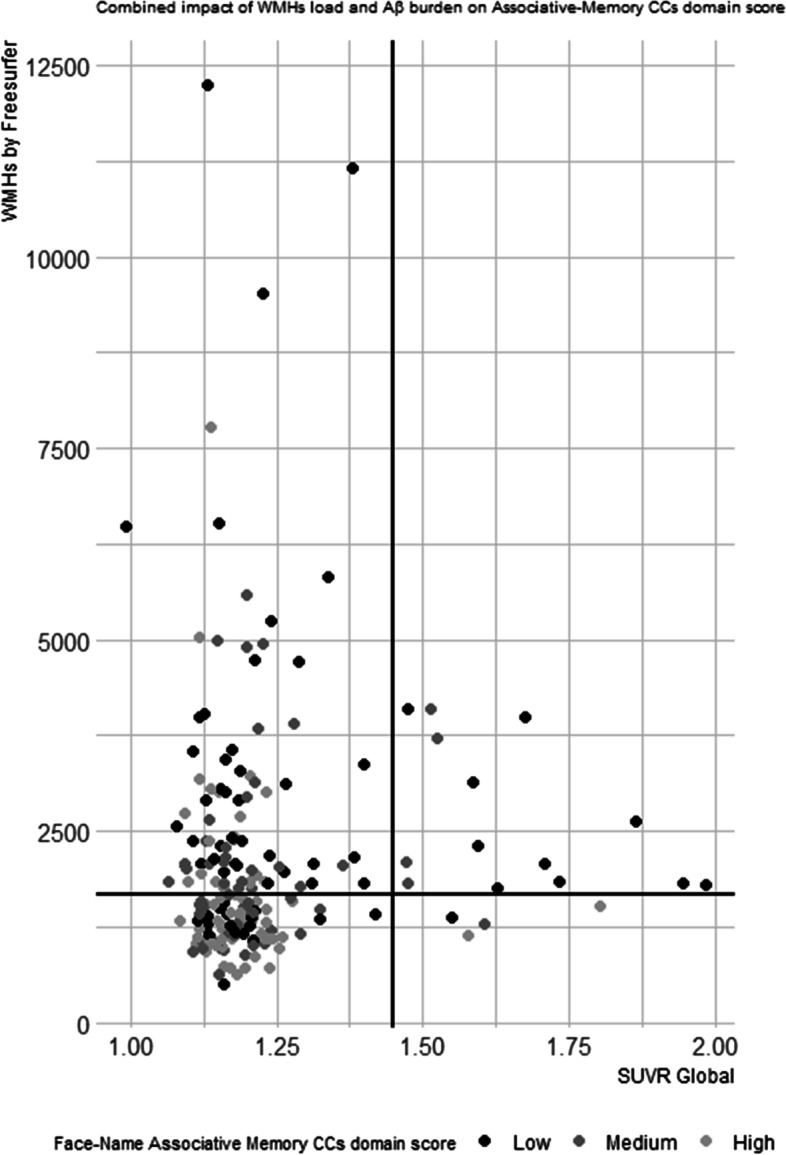
Combined impact of WMH load and Aß burden face–name associative-memory CCs domain score. The SCD subjects with high performances on the face–name associative memory CCs domain score are shown with lighter color intensity dots, those with medium performances are represented with medium color intensity dots, while those with low performances are shown with darker color intensity dots

However, FreeSurfer (*B* =  − 1.095, 95% CI =  − 1.729, − 0.461, *p* = 0.001) and Aβ deposition (SUVR) (*B* =  − 1.320, 95% CI =  − 2.222, − 0.418, *p* = 0.004) were independently associated with this CCs domain score. Thus, a cumulative effect of the combination of these two pathological conditions promotes a lower cognitive performance. The distribution of face–name associative memory CCs domain score performance according to WMHs (FreeSurfer) and Aβ deposition is reported in Fig. 1 graphically. In this figure, because of the fact that the white matter hypointensities load measured by the FreeSurfer pipeline has no published cutoff scores, this variable was dichotomized, generating the categories high WMHs (H-WMHs) and low WMHs (L-WMHs), taking its median value as a cutoff (Md = 1675.25). Raw units were used for this purpose only. We can see how those individuals with either Aβ + or Aβ − and high load of white matter hyperintensities (H-WMHs) showed poorer performance on the CCs domain of face–name associative memory, particularly those with H-WMHs and higher Aβ deposition.

## Discussion

In this cross-sectional study, we analyzed whether WMHs and Aβ burden were associated with cognitive performance on specific optimized CCs domain scores in cognitively healthy individuals with SCD from the FACEHBI cohort [14]. Our data identified a positive association of increased WMH load measured by FreeSurfer and poor episodic memory performance and a tendency between increased Aβ burden (SUVR) with a worse episodic memory performance in SCD individuals. Although there was no modulation effect of these two pathological conditions on the face–name associative memory CCs domain score, its cumulative effects according to both pathologies indicated a worse performance on the same CCs domain.

Given the clinical relevance of WMHs, and that individuals with severe WMHs have a fourfold higher risk of showing clinical progression to mild cognitive impairment or dementia, as compared with those without WMHs [32], methods for reliably classifying the severity of WMH load are useful not only in clinical practice but also for research. Despite their great utility, visual ratings of WMHs such as the Fazekas scale or the ARWMC scale are subjective, which often compromises inter-rater reliability. Automated volumetric quantification of brain vascular pathology based on MRI measures, such as the FreeSurfer pipeline, is considered to be an applicable method [33] and has been shown to be comparable in accuracy to manual labeling for many functions [34]. FreeSurfer is an image analysis suite, well documented and freely available for download online. The fact that FreeSurfer is a standalone package that does not depend on any commercial license allows to reproduce our results easily for anyone. While it has been found that WMH lesions could produce systematic errors in FreeSurfer GM segmentations, this fact does not imply that WMHs are wrong measures; we should take into account that subjects analyzed along this work present low WMH damage. Moreover, Hotz et al. [24] compares three algorithms for the measure of the WMH and find that “FreeSurfer fundamentally underestimated the WMHs volume in comparison with the gold standard” but also that “its WMHs volumes correlated strongly with the Fazekas scores and showed no conspicuous WMH volume increases and decreases between measurement points in the longitudinal data” that, from our point of view, makes it more reliable for the kind of study conducted here. Thus, this approach allowed us to compare the results obtained using each of these scales in relation to different variables.

Consistent with studies published previously [35–37], we found an association between higher WMH load and worse performance on episodic memory tasks, in our case measured by FreeSurfer. Our findings confirmed the relationship between WMH load and the first signs of memory loss or poor cognitive performance, as other research studies have demonstrated [32, 38, 39] with WMH load being detectable decades before clinical symptoms of cognitive impairment appear. These associations were independent of age, sex, education, and *APOE* status, factors that over the years have been indicated as being strong predictors of cognitive impairment and dementia. Therefore, as Marije et al. [32] noted, our results suggest that measures of WMHs in individuals exhibiting no objective cognitive symptoms except for SCD and poor baseline cognitive performance could reflect an early stage of actual cognitive decline. Nevertheless, to our knowledge, our study is the first to use and report associations between these three neuroimaging scales assessing WMH load and cognitive performance in SCD individuals.

Subjective cognitive decline has been demonstrated to be a risk factor for progression to AD dementia in cognitively healthy elderly individuals [40, 41]. Consistent with previous cross-sectional studies [25, 42], in the current study, although we did not find a statistical significance, a tendency between poorer performance on the CCs domain of face–name associative memory was found to be related to higher brain Aβ deposition in cognitively healthy individuals. Therefore, we consider this finding clinically important and warrant further consideration. What is unique in our study, however, is that we investigated a possible additive effect of the combination of Aβ deposition and WMH load in SCD subjects.

Given the results obtained and bearing in mind the relationship with vascular brain tissue damage, such as WMH load and amyloid status [7–9], one of our hypotheses was that a possible additive effect of WMH load and Aβ burden combined could result in poorer cognitive performance. Although there was no interaction effect of these two pathological conditions on face–name associative memory CCs domain score, its cumulative effects according to both pathologies indicated a worse performance on the same CCs domain. Further work is needed to demonstrate whether this additive or synergistic effect is sustained over time. As a matter of fact, one of our main findings, like those of previous studies [43], reinforces the relevance of the CCs domain score of face–name associative memory as a promising neurocognitive endophenotype in the detection of the combination of Aβ burden and WMH load in individuals with SCD.

We acknowledge that the present study has a relatively low prevalence of brain vascular damage in the sample; therefore, the findings presented here need to be interpreted cautiously and require further follow-up. Moreover, the use of 1.5 T could be considered as a limitation of the study since the lesion detection could be underestimated when compared with a 3-T scanner. The conjunction of a 1.5-T scanner and low vascular damage is not the ideal scenario for WMH studies even when a T2 3D FLAIR is used. Additionally, the cross-sectional nature of this study allows assumptions regarding associations to be made, but does not allow causal inferences. However, this study did include the use of detailed CCs domain scores and high-quality assessment of WMH load through three different neuroimaging scales, and as we excluded participants with strokes and dementia, the use of a relatively healthy group allowed us to “isolate” as much as possible the cognitive and anatomical effects of vascular risk factors. Finally, our results are in line with previous findings [32, 44], in which a heterogeneous population of nondisabled elderly people with WMHs was included [44]. We are, however, the first to show the effect of the cumulative effect of WMHs and Aβ burden in subjects presenting with SCD in a clinical setting.

## Conclusions

Our results suggest that increased WMH load and increased Aβ are independently associated with poorer episodic memory performance in SCD individuals, indicating a cumulative effect of the combination of these two pathological conditions in promoting lower cognitive performance, an aspect that could help in terms of treatment and prevention.

### Limitations

We acknowledge that the present study has a relatively low prevalence of brain vascular damage in the sample; therefore, the findings presented here need to be interpreted cautiously and require further follow-up. Moreover, the use of 1.5 T could be considered as a limitation of the study since the lesion detection could be underestimated when compared with a 3-T scanner. The conjunction of a 1.5-T scanner and low vascular damage is not the ideal scenario for WMH studies even when a T2 3D FLAIR is used. Additionally, the cross-sectional nature of this study allows assumptions regarding associations to be made, but does not allow causal inferences.

## Data Availability

Data used can be requested through the corresponding author.

## Abbreviations

AD: Alzheimer’s disease
*APOE*: Apolipoprotein E
ARWMC: Age-Related White Matter Changes
Aβ: Amyloid-beta
CVD: Cerebrovascular disease
CCs: Cognitive composites
DWM: Deep white matter
DM: Diabetes mellitus
DLP: Hyperlipidemia
FACEHBI: Fundació ACE Brain Health Initiative
FBB-PET: ^18^F-florbetaben positron emission tomography
H-WMHs: High load of white matter hyperintensities
HDL-C: High-density lipoprotein cholesterol
L-WMHs: Low load of white matter hyperintensities
MCI: Mild cognitive impairment
MRI: Magnetic resonance imaging
OB: Obesity/overweight
PCA: Principal component analysis
PVWM: Periventricular white matter
SCD: Subjective cognitive decline
SUVR: Standardized uptake value ratio
SVD: Small vessel disease
VCI: Vascular cognitive impairment
VRFs: Cardio-metabolic risk factors
WC: Waist circumference
WMHs: White matter hyperintensities
